# Merging of Numerical Intervals in Entropy-Based Discretization

**DOI:** 10.3390/e20110880

**Published:** 2018-11-16

**Authors:** Jerzy W. Grzymala-Busse, Teresa Mroczek

**Affiliations:** 1 Department of Electrical Engineering and Computer Science, University of Kansas, Lawrence, KS 66045, USA; 2 Department of Expert Systems and Artificial Intelligence, University of Information Technology and Management, Rzeszow 35-225, Poland

**Keywords:** data mining, discretization, numerical attributes, entropy

## Abstract

As previous research indicates, a multiple-scanning methodology for discretization of numerical datasets, based on entropy, is very competitive. Discretization is a process of converting numerical values of the data records into discrete values associated with numerical intervals defined over the domains of the data records. In multiple-scanning discretization, the last step is the merging of neighboring intervals in discretized datasets as a kind of postprocessing. Our objective is to check how the error rate, measured by tenfold cross validation within the C4.5 system, is affected by such merging. We conducted experiments on 17 numerical datasets, using the same setup of multiple scanning, with three different options for merging: no merging at all, merging based on the smallest entropy, and merging based on the biggest entropy. As a result of the Friedman rank sum test (5% significance level) we concluded that the differences between all three approaches are statistically insignificant. There is no universally best approach. Then, we repeated all experiments 30 times, recording averages and standard deviations. The test of the difference between averages shows that, for a comparison of no merging with merging based on the smallest entropy, there are statistically highly significant differences (with a 1% significance level). In some cases, the smaller error rate is associated with no merging, in some cases the smaller error rate is associated with merging based on the smallest entropy. A comparison of no merging with merging based on the biggest entropy showed similar results. So, our final conclusion was that there are highly significant differences between no merging and merging, depending on the dataset. The best approach should be chosen by trying all three approaches.

## 1. Introduction

Discretization of numerical attributes is an important technique used in data mining. Discretization is the process of converting numerical values of data records into discrete values associated with numerical intervals defined over the domains of the data records. As is well known, discretization based on entropy is very successive [1,2,3,4,5,6,7,8,9,10,11,12,13,14,15,16,17,18,19,20,21,22,23,24,25,26]. Additionally, many new techniques have been proposed, e.g., discretization using statistical and logical analysis of data [27], discretization using low-frequency values and attribute interdependency [28], discretization based on rough-set theory [29], a hybrid scheme of frequency and expected number of so-called segments of examples [30], and an oversampling technique combined with randomized filters [31]. Entropy-based discretization was also used for special purposes, e.g., for ranking [32] and for stock-price forecasting [33].

As follows from recent research [13,34,35], one of the discretization methods, called multiple scanning and based on entropy, is especially successful. An important step of such discretization is merging intervals, conducted as the last step of discretization. As a result, some pairs of intervals are replaced by new, larger intervals. In this paper, we compare two methods of merging numerical intervals, based on the smallest and biggest entropy by skipping merging, i.e., no merging at all. Our results show that such interval merging is crucial for quality of discretization.

The multiple-scanning discretization method, as the name indicates, is based on scanning the entire set of attributes many times. During every scan, for every attribute, the best cutpoint is identified. The quality of a cutpoint is estimated by the conditional entropy of the decision given an attribute. The best cutpoint is associated with the smallest conditional entropy. For a specific scan, when all best cutpoints are selected, a set of subtables is created; each such subtable needs additional discretization. Every subtable is scanned again, and the best cutpoints are computed. There are two ways to end this process: either the stopping condition is satisfied, or the requested number of scans is achieved. If the stopping condition is not satisfied, discretization is completed by another discretization method called Dominant Attribute [34,35].

Dominant-attribute discretization uses a different strategy than multiple scanning, but it is also using many step approach to discretization. In every step, first the best attribute is selected by using the minimum of the conditional entropy of decision given attribute condition. Then, the best cutpoint is identified using the same principle. Discretization is complete when the stopping condition is satisfied.

The multiple-scanning methodology is better than two well-known discretization methods: Equal Interval Width and Equal Frequency per Interval enhanced to globalized methods [34]. In Reference [34], rule induction was used for data mining. Additionally, four other discretization methods, namely, the original C4.5 approach to discretization, and the same globalized versions of Equal Interval Width and Equal Frequency per Interval methods, and Multiple Scanning were compared in Reference [35]; this time, data mining was based on the C4.5 generation of decision trees. Again, it was shown that the best discretization method is Multiple Scanning.

## 2. Discretization

Let *a* be a numerical attribute, $a_{i}$ be the smallest value of *a*, and $a_{j}$ be the largest value of *a*. Discretization of *a* is based on finding the numbers $a_{i_{0}}$, $a_{i_{1}}$, …, $a_{i_{k}}$, called cutpoints, where $a_{i_{0}}=a_{i}$, $a_{i_{k}}=a_{j}$, $a_{i_{l}}$ < $a_{i_{l+1}}$ for *l* = 0, 1, …, k−1, and *k* is a positive integer. Thus, domain $\left[a_{i},a_{j}\right]$ of *a* is partitioned into *k* intervals

$$\{\left[a_{i_{0}},a_{i_{1}}\right),\left[a_{i_{1}},a_{i_{2}}\right),\ldots ,\left[a_{i_{k-2}},a_{i_{k-1}}\right),\left[a_{i_{k-1}},a_{i_{k}}\right]\}.$$

In the remainder of this paper, such intervals are denoted as follows:

$$a_{i_{0}}\ldots a_{i_{1}},a_{i_{1}}\ldots a_{i_{2}},\ldots ,a_{i_{k-2}}\ldots a_{i_{k-1}},a_{i_{k-1}}\ldots a_{i_{k}}.$$

In practical applications, discretization is conducted on many numerical attributes. Table 1 presents an example of a dataset with four numerical attributes: Length, Height, Width, and Weight, and eight cases. An additional symbolic variable, Quality, is the decision. Attributes are independent variables, while the decision is a dependent variable. The set of all cases is denoted by *U*. In Table 1, *U* = {1, 2, 3, 4, 5, 6, 7, 8}.

Let *v* be a variable and let $v_{1}$, $v_{2}$, …, $v_{n}$ be values of *v*, where *n* is a positive integer. Let *S* be a subset of *U*. Let $p(v_{i})$ be a probability of $v_{i}$ in *S*, where *i* = 1, 2, …, n. An *entropy* $H_{S}\left(v\right)$ is defined as follows:

$$H_{S}\left(v\right)=-\sum_{i=1}^{n}p\left(v_{i}\right)\cdot \log p\left(v_{i}\right).$$

In this paper, we assume that all logarithms are binary.

Let *a* be an attribute, let $a_{1}$, $a_{2}$, …, $a_{m}$ be all values of *a* restricted to *S*, let *d* be a decision and let $d_{1}$, $d_{2}$, …, $d_{n}$ be all values of *d* restricted to *S*. Conditional entropy $H_{S}\left(d|a\right)$ of the decision *d* given attribute *a* is defined as follows:

$$-\sum_{j=1}^{m}p\left(a_{j}\right)\cdot \sum_{i=1}^{n}p\left(d_{i}|a_{j}\right)\cdot log\;p\left(d_{i}|a_{j}\right),$$

where $p(d_{i}|a_{j})$ is the conditional probability of the value $d_{j}$ of the decision *d* given $a_{j}$; j∈{1,2,…,m} and i∈{1,2,…,n}.

As is well known [1,4,5,7,9,10,12,13,16,21,23,24], discretization that uses conditional entropy of the decision-given attribute is believed to be one of the most successful discretization techniques.

Let *S* be a subset of *U*, *a* be an attribute, and *q* be a cutpoint splitting the set *S* into two subsets, $S_{1}$ and $S_{2}$. The corresponding conditional entropy, denoted by $H_{S}\left(d|a\right)$ is defined as follows:

$$\frac{|S_{1}|}{\left|U\right|}H_{S_{1}}\left(a\right)+\frac{|S_{2}|}{\left|U\right|}H_{S_{2}}\left(a\right),$$

where |X| denotes the cardinality of set *X*. Usually, cutpoint *q* for which $H_{S}\left(d|a\right)$ is the smallest is considered to be the best cutpoint.

We need a condition to stop discretization. Roughly speaking, the most obvious idea is to stop discretization when we may distinguish the same cases in the discretized dataset that were distinguishable in the original dataset with numerical attributes. The idea of distinguishability (indiscernibility) of cases is one of the basic ideas of rough-set theory [36,37]. Let *B* be a subset of set *A* of all attributes, and x,y∈U. Indiscernibility relation *IND*(B) is defined as follows:

(x,y)∈IND(B) if and only if a(x)=a(y) for any a∈B,

where a(x) denotes the value of the attribute a∈A for the case x∈U. Obviously, IND(B) is an equivalence relation. For x∈U, the equivalence class of IND(B) is denoted by ${\left[x\right]}_{B}$, and is called a B-elementary set.

A family of all sets ${\left[x\right]}_{B}$, where x∈U, is a partition on *U*, denoted by $B^{*}$. Additionally, for a decision *d*, a ${\left\{d\right\}}^{*}$-elementary set is called a *concept*. For Table 1, and for *B* = {*Length*}, $B^{*}$ = {{1, 3}, {2, 4, 7, 8}, {5, 6}} and {${d\}}^{*}$ = {{1, 2, 3}, {4, 5}, {6}, {7, 8}}. None of the concepts {1, 2, 3}, {4, 5}, {6}, {7, 8} is *B*-definable. It is a usual practice in rough-set theory to use for any $X\in {\left\{d\right\}}^{*}$ two sets, called lower and upper approximations of *X*. The lower approximation of *X* is defined as follows:

$$\left\{x\;|\;x\in U,{\left[x\right]}_{B}\subseteq X\right\}$$

and is denoted by $\underline{B}X$. The upper approximation of *X* is defined as follows:

$$\left\{x\;|\;x\in U,{\left[x\right]}_{B}\cap X\neq \emptyset \right\}$$

and is denoted by $\bar{B}X$. For Table 1, $\underline{B}${1, 2, 3} = {1, 3} and $\bar{B}${1, 2, 3} = {1, 2, 3, 4, 7, 8}.

Usually, discretization is stopped when so-called level of consistency [4], defined as follows:

$$L\left(A\right)=\frac{\sum_{X\in {\left\{d\right\}}^{*}}\left|\underline{A}X\right|}{\left|U\right|}$$

and denoted by L(A), is equal to 1. For Table 1, $A^{*}$ = {{1}, {2}, {3}, {4}, {5}, {6}, {7}, {8}}, so $\underline{A}X$ = *X* for any concept *X* from ${\left\{d\right\}}^{*}$. On the other hand, for *B* = {*Length*},

$$L\left(B\right)=\frac{|\underline{B}\left\{1,2,3\right\}|+|\underline{B}\left\{4,5\right\}|+|\underline{B}\left\{6\right\}|+|\underline{B}\left\{7,8\right\}|}{\left|U\right|}=\frac{\left|\{1,3\}|+|\emptyset |+|\emptyset |+|\emptyset |+|\emptyset \right|}{8}=0.25.$$

### 2.1. Multiple Scanning

Special parameter *t*, selected by the user and called the total number of scans, is used in multiple-scanning discretization. During the first scan, for any attribute *a* from the set *A*, the best cutpoint is selected using the criterion of smallest entropy $H_{U}\left(d|q\right)$ for all potential cutpoints splitting *U*, where *d* is the decision. Such cutpoints are created as the averages of two consecutive values of sorted attribute *a*. Once the best cutpoint is found, a new binary attribute $a^{d}$ is created, with two intervals as vales of $a^{d}$, the first interval is defined as containing all original numerical values of *a* smaller than the selected cutpoint *q*, and the second interval contains the remaining original values of *a*. Partition ${\left\{A^{d}\right\}}^{*}$ is created, where $A^{d}$ is the set of all partially discretized attributes. For the next scans, starting from *t* = 2, set *A* is scanned again: for each block *X* of ${\left\{A^{d}\right\}}^{*}$, for each attribute *a*, and for each remaining cutpoint of *a*, the best cutpoint is computed, and the best cutpoint among all blocks *X* of ${\left\{A^{d}\right\}}^{*}$ is selected as the next cutpoint of *a*. If parameter *t* is reached and $L(A^{d})\neq 1$, another discretization method, Dominant Attribute, is used. In the dominant-attribute strategy, the best attribute is first selected among partially discretized attributes, using the criterion of smallest conditional entropy $H(d|a^{d})$, where $a^{d}$ is a partially discretized attribute. For the best attribute, best cutpoint *q* is selected, using the criterion of smallest entropy $H_{S}\left(d|a^{d}\right)$, where *q* splits *S* into $S_{1}$ and $S_{2}$. For both $S_{1}$ and $S_{2}$, we select the best attribute and then the best cutpoint, until $L(A^{d})=1$, where $A^{d}$ is the set of discretized attributes.

We illustrate the multiple-scanning discretization method using the dataset from Table 1. Since our dataset was small, we used just one scan. Initially, for any attribute a∈A, all conditional entropies $H_{a}\left(q,U\right)$ should be computed for all possible cutpoints *q* of *a*. The set of all possible cutpoints for Length is {4.4, 4.6}. Similarly, the sets of all possible cutpoints for Height, Width, and Weight were {1.5, 1.7}, {1.75, 1.85} and {1.1, 1.5}, respectively. Furthermore,

$$H_{Length}\left(4.4,U\right)=\frac{2}{8}\left(-\frac{1}{2}\cdot log\frac{1}{2}\right)2+\frac{6}{8}\left(-\frac{3}{6}\cdot log\frac{3}{6}-\frac{2}{6}\cdot log\frac{2}{6}-\frac{1}{6}\cdot log\frac{1}{6}\right)=1.344,$$

$$H_{Length}\left(4.6,U\right)=\frac{6}{8}\left(\left(-\frac{2}{6}\cdot log\frac{2}{6}\right)2+\left(-\frac{1}{6}\cdot log\frac{1}{6}\right)2\right)+\frac{2}{8}\left(0\right)=1.439.$$

The best cutpoint is 4.4. In a similar way, we selected the best cutpoints for the remaining attributes, Height, Width, and Weight. These cutpoints are 1.5, 1.75, and 1.1, respectively. Thus, the partially discretized dataset, after the first scan, is presented in Table 2.

The dataset from Table 2 needs an additional discretization since ${\left(A^{d}\right)}^{*}$ = {{1, 4}, {2}, {3}, {5}, {6}, {7}, {8}}, ${\left\{d\right\}}^{*}$ = {{1, 2, 3}, {4, 5}, {6}, {7, 8}} and

$$L\left(\left\{Length^{d},\;Height^{d},\;Width^{d},\;Weigth^{d}\right\}\right)=\frac{2+1+1+2}{8}=0.75<1.$$

As follows from Table 2, Cases 1 and 4 need to be distinguished. A dataset from Table 1, restricted to Cases 1 and 4, is presented in Table 3.

Cases 1 and 4 from Table 3 may be distinguished by any of the two following attributes: Length and Weight. Both attributes are of the same quality, as a result of a heuristic step we selected Length. A new cutpoint for Length was equal to 4.6. Thus, attribute Length has two cutpoints, 4.4 and 4.6. Table 4 presents the next partially discretized dataset.

For the dataset from Table 4, ${\left(A^{d}\right)}^{*}=\left\{\left\{1\right\},\left\{2\right\},\left\{3\right\},\left\{4\right\},\left\{5\right\},\left\{6\right\},\left\{7\right\},\left\{8\right\}\right\}$ and L(A) = 1.

### 2.2. Interval Merging

In general, it is possible to simplify the result of discretization by interval merging. The idea is to replace two neighboring intervals, i…j and j…k, of the same attribute by one interval, i…k. It can be conducted using two different techniques: safe merging and proper merging. In safe merging, for a given attribute, any two neighboring intervals i…j and j…k are replaced by interval i…k, if for both intervals the decision value is the same.

In proper merging, two neighboring intervals i…j and j…k of the same attribute are replaced by interval i…k, if the levels of consistency before merging and after merging are the same. A question is how to guide the search for such two neighboring intervals. In experiments described in this paper, two search criteria were implemented based on the smallest and the largest conditional entropy $H_{S}\left(d|a\right)$. Another possibility, also taken into account, is ignoring any merging at all.

It is clear that, for Table 4, for the Length attribute, we may eliminate Cutpoint 4.4. As a result, a new data set, presented in Table 5 is created. For the dataset from Table 4, ${\left(A^{d}\right)}^{*}=\left\{\left\{1\right\},\left\{2\right\},\left\{3\right\},\left\{4\right\},\left\{5\right\},\left\{6\right\},\left\{7\right\},\left\{8\right\}\right\}$ and L(A) = 1.

## 3. Experiments

Experiments described in this paper were conducted on 17 datasets with numerical attributes. These datasets presented in Table 6 and are accessible in the Machine-Learning Repository, University of California, Irvine, except for bankruptcy. The bankruptcy dataset was given in Reference [38].

For discretization, we applied the multiple-scanning method. The level of consistency was set to 1. We used three approaches to merging intervals in the last stage of discretization:

- no merging at all,
- proper merging based on the minimum of conditional entropy, and
- proper merging based on the maximum of conditional entropy.

The discretized datasets were processed by the C4.5 decision-tree generating system [39]. Note that the C4.5 system builds a decision tree using conditional entropy as well. The main mechanism of selecting the most important attribute *a* in C4.5 is based on the maximum of mutual information, which in C4.5 is called an information gain. The mutual information is the difference between marginal entropy $H_{S}\left(d\right)$ and conditional entropy $H_{S}\left(d|a\right)$, where *d* is the decision. Since $H_{S}\left(d\right)$ is fixed, the maximum of mutual information is equivalent to the minimum of conditional entropy $H_{S}\left(d|a\right)$. In our experiments, an error rate was computed using internal tenfold cross validation of C4.5.

**Figure 1:**
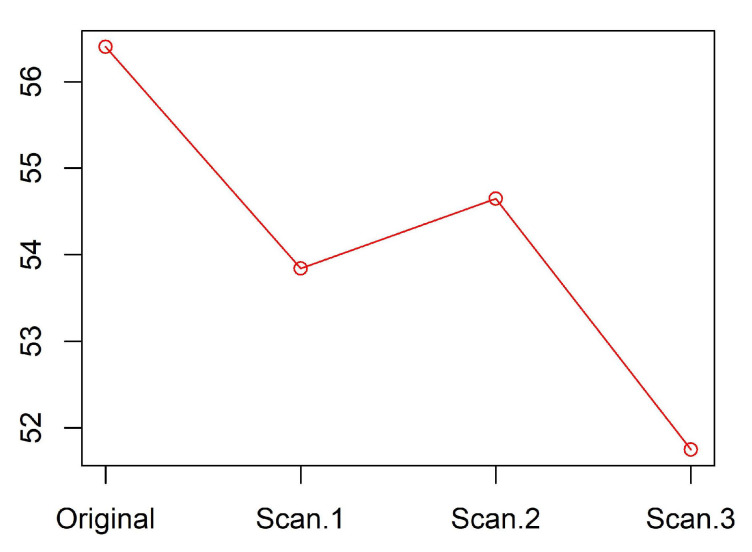
Error rate for consecutive scans for the yeast dataset.

**Figure 2:**
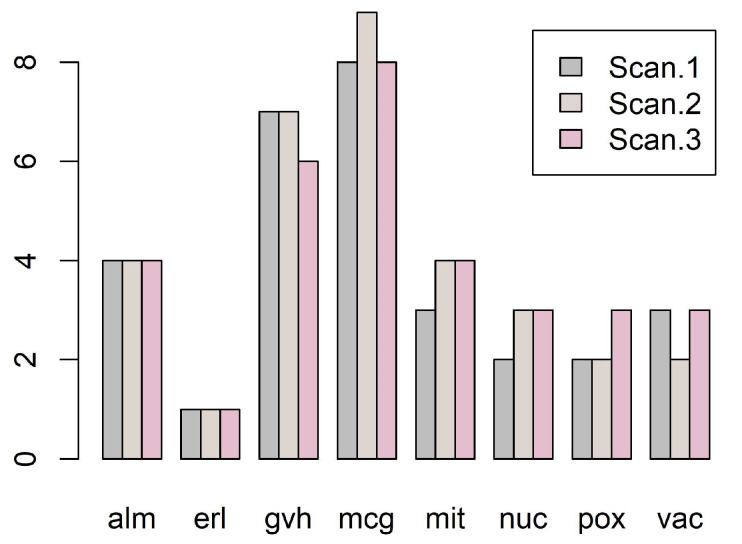
Number of discretization intervals for consecutive scans for the yeast dataset.

**Figure 3:**
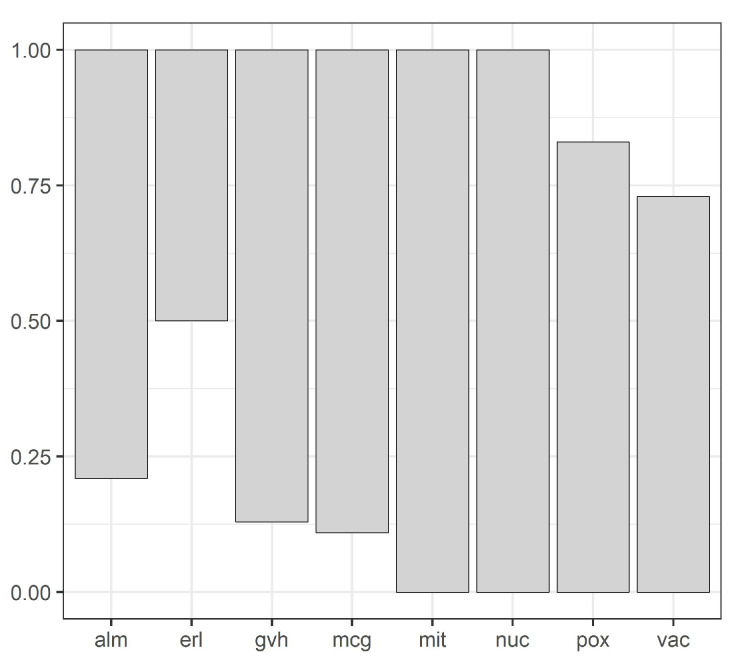
Domains of all attributes for the yeast dataset.

**Figure 4:**
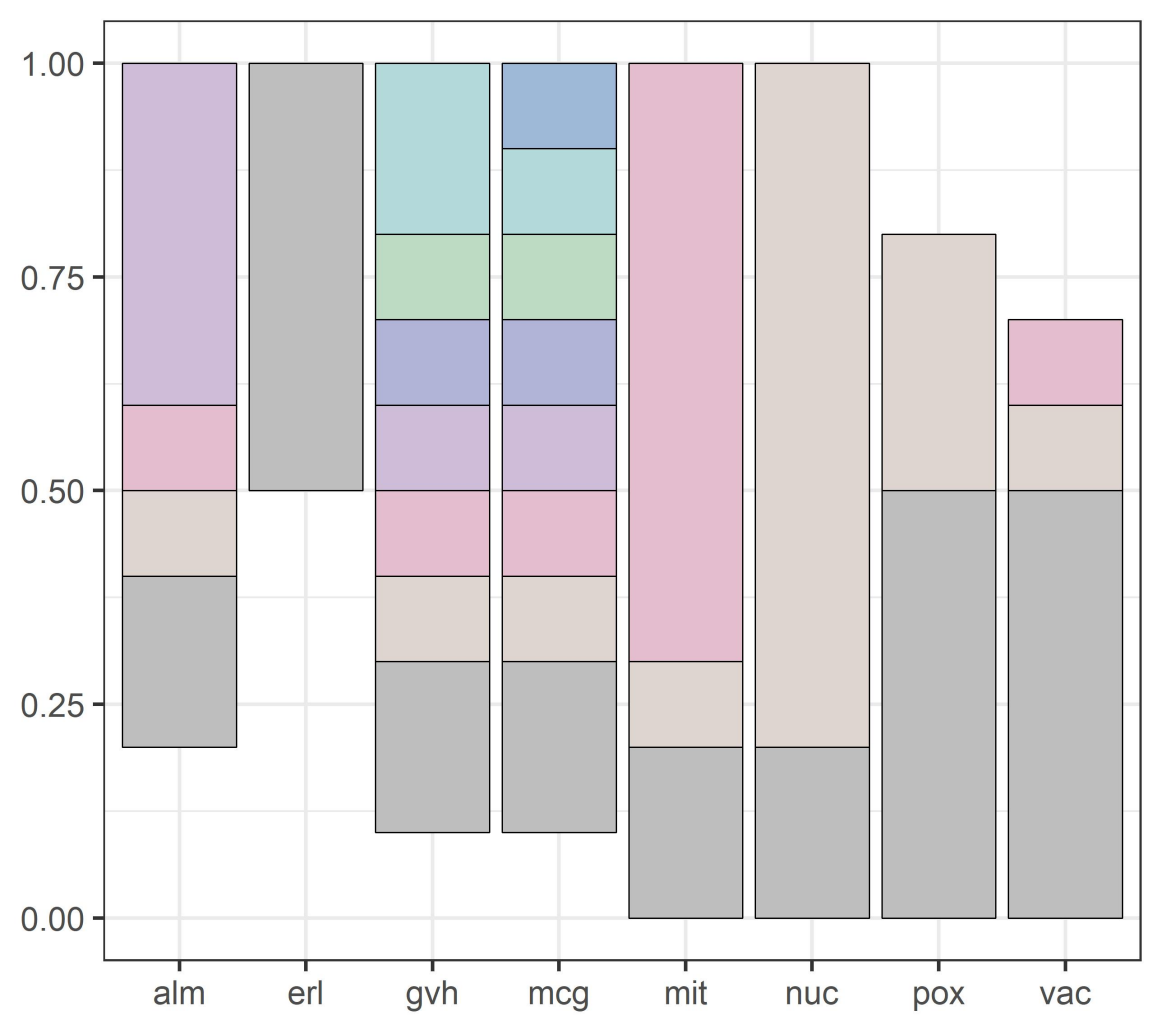
Intervals for all attributes after the first scan for the yeast dataset.

**Figure 5:**
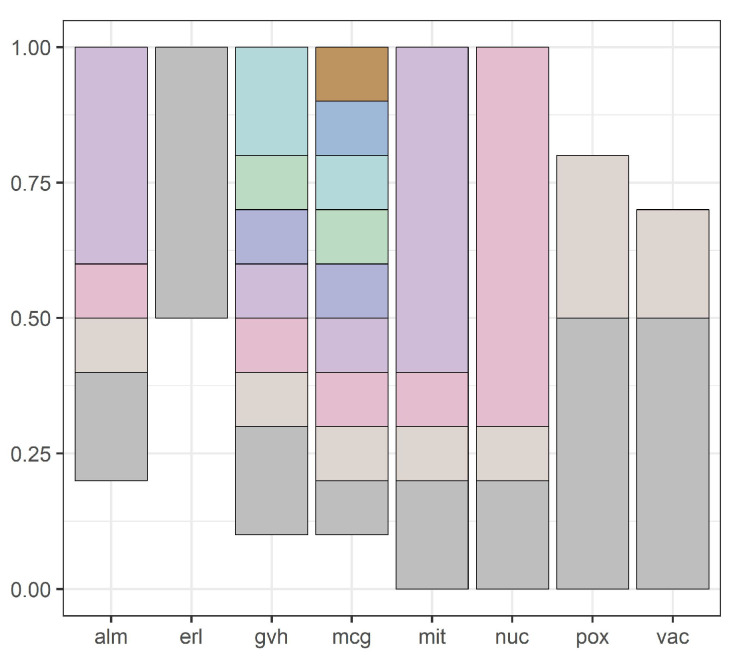
Intervals for all attributes after the second scan for the yeast dataset.

**Figure 6:**
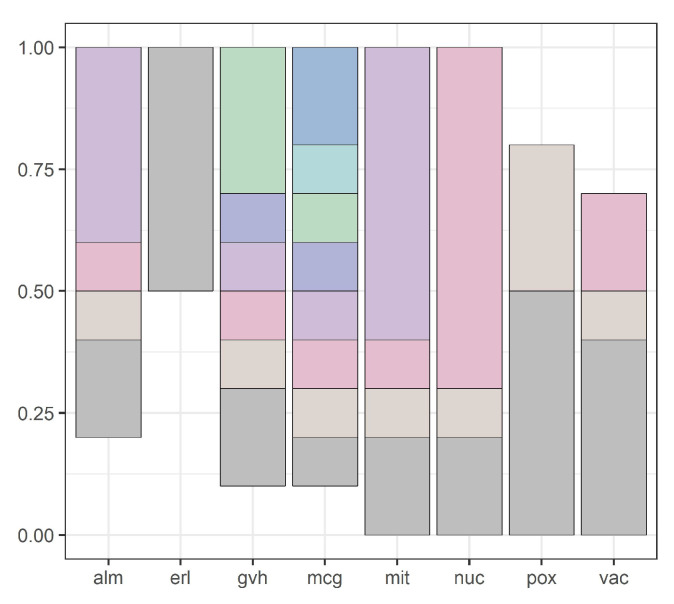
Intervals for all attributes after the third scan for the yeast dataset.

**Figure 7:**
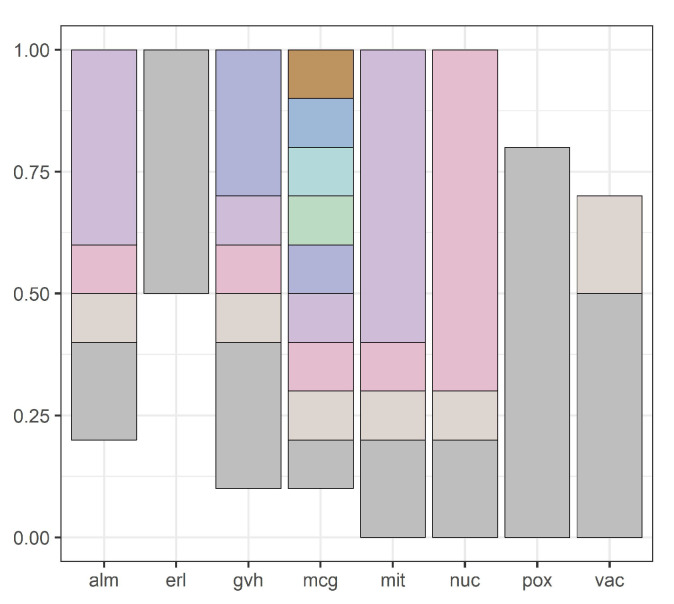
Intervals for all attributes after merging based on minimal entropy for the second scan for the yeast dataset.

**Figure 8:**
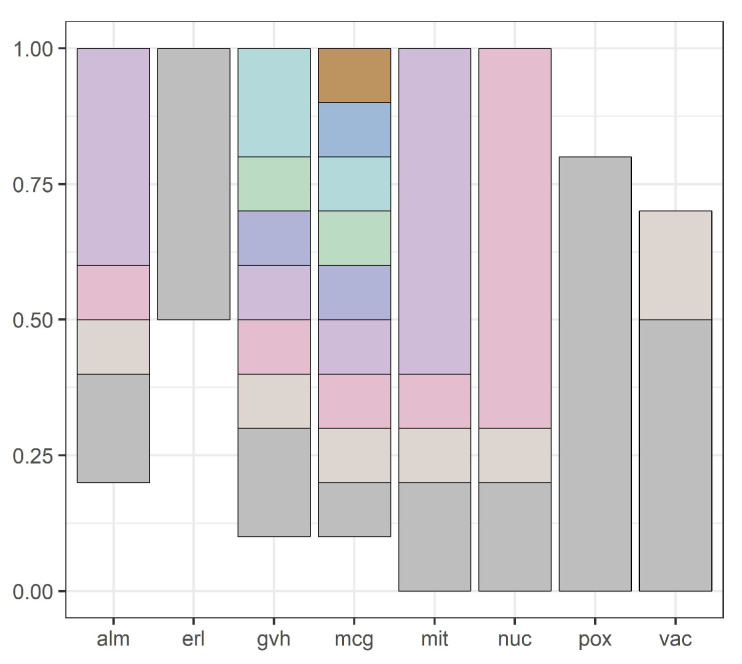
Intervals for all attributes after merging based on maximal entropy for the second scan for the yeast dataset.

Our methodology is illustrated by Figure 1, Figure 2, Figure 3, Figure 4, Figure 5, Figure 6, Figure 7 and Figure 8, all restricted to the yeast dataset, one of 17 datasets used for experiments. Figure 1 presents an error rate for three consecutive scans conducted on the yeast dataset. Figure 2 shows the number of discretization intervals for three scans on the same dataset. Figure 3 shows domains of all attributes for the yeast dataset, and Figure 4, Figure 5, Figure 6, Figure 7 and Figure 8 show intervals of all attributes during interval scanning and merging.

Table 7 shows error rates for the three approaches to merging. Note that, for any dataset, we included only the smallest error rate with a corresponding scan number. The error rates were compared using the Friedman rank sum test combined with multiple comparison, with 5% level of significance. As follows from the Friedman test, the differences between all three approaches are statistically insignificant.

Thus, there is no universally best approach among no merging, merging based on minimum of conditional entropy, and merging based on maximum of conditional entropy.

Our next objective was to test the difference between all three approaches for a specific dataset. We conducted extensive experiments, with the repetition of 30 tenfold cross validations for every dataset and recorded averages and standard deviations in order to use the standard test for difference between averages. The corresponding *Z* scores are presented in Table 8. It is quite obvious that the choice of the correct approach to merging is highly significant in most cases, with the level of significance at 0.01, since the absolute value of the corresponding *Z*-score is larger than 2.58. For example, for the ecoli dataset, merging of intervals based on minimum of conditional entropy is better than no merging, while for the leukemia dataset, it is the other way around. Similarly, for the ecoli dataset, no merging is better than merging based on the maximum of conditional entropy, while for the pima dataset it is the opposite.

Our future research plans include a comparison of our main methodology, multiple-scanning discretization, with discretization based on binning using histograms and chi-square analysis.

## 4. Conclusions

The main contribution of our paper is showing that postprocessing discretization based on merging intervals is extremely important for the discretization quality. Results of our experiments indicate that there is no universally best approach to merging intervals. However, there are statistically highly significant differences (with 1% significance level) between these three approaches, depending on the dataset. Therefore, it is very important to use the best choice among the three approaches during multiple-scanning discretization of datasets with numerical attributes.

## Figures and Tables

**Table 1:** An example of a dataset with numerical attributes.

Case	Attributes				Decision
	Length	Height	Width	Weight	Quality
1	4.7	1.8	1.7	1.7	high
2	4.5	1.4	1.8	0.9	high
3	4.7	1.8	1.9	1.3	high
4	4.5	1.8	1.7	1.3	medium
5	4.3	1.6	1.9	1.7	medium
6	4.3	1.4	1.7	0.9	low
7	4.5	1.6	1.9	0.9	very-low
8	4.5	1.4	1.8	1.3	very-low

**Table 2:** Partially discretized dataset after the first scan. (${}^{d}$ is the decision)

Case	Attributes				Decision
	Length ${}^{d}$	Height ${}^{d}$	Width ${}^{d}$	Weight ${}^{d}$	Quality
1	4.4..4.7	1.5..1.8	1.7..1.75	1.1..1.7	high
2	4.4..4.7	1.4..1.5	1.75..1.9	0.9..1.1	high
3	4.4..4.7	1.5..1.8	1.75..1.9	1.1..1.7	high
4	4.4..4.7	1.5..1.8	1.7..1.75	1.1..1.7	medium
5	4.3..4.4	1.5..1.8	1.75..1.9	1.1..1.7	medium
6	4.3..4.4	1.4..1.5	1.7..1.75	0.9..1.1	low
7	4.4..4.7	1.5..1.8	1.75..1.9	0.9..1.1	very-low
8	4.4..4.7	1.4..1.5	1.75..1.9	1.1..1.7	very-low

**Table 3:** A subset of the dataset presented in Table 1.

Case	Attributes				Decision
	Length	Height	Width	Weight	Quality
1	4.7	1.8	1.7	1.7	high
4	4.5	1.8	1.7	1.3	medium

**Table 4:** Discretized dataset.

Case	Attributes				Decision
	Length ${}^{d}$	Height ${}^{d}$	Width ${}^{d}$	Weight ${}^{d}$	Quality
1	4.6..4.7	1.5..1.8	1.7..1.75	1.1..1.7	high
2	4.4..4.6	1.4..1.5	1.75..1.9	0.9..1.1	high
3	4.6..4.7	1.5..1.8	1.75..1.9	1.1..1.7	high
4	4.4..4.6	1.5..1.8	1.7..1.75	1.1..1.7	medium
5	4.3..4.4	1.5..1.8	1.75..1.9	1.1..1.7	medium
6	4.3..4.4	1.4..1.5	1.7..1.75	0.9..1.1	low
7	4.4..4.6	1.5..1.8	1.75..1.9	0.9..1.1	very-low
8	4.4..4.6	1.4..1.5	1.75..1.9	1.1..1.7	very-low

**Table 5:** Discretized dataset after interval merging.

Case	Attributes				Decision
	Length ${}^{d}$	Height ${}^{d}$	Width ${}^{d}$	Weight ${}^{d}$	Quality
1	4.6..4.7	1.5..1.8	1.7..1.75	1.1..1.7	high
2	4.3..4.6	1.4..1.5	1.75..1.9	0.9..1.1	high
3	4.6..4.7	1.5..1.8	1.75..1.9	1.1..1.7	high
4	4.3..4.6	1.5..1.8	1.7..1.75	1.1..1.7	medium
5	4.3..4.4	1.5..1.8	1.75..1.9	1.1..1.7	medium
6	4.3..4.6	1.4..1.5	1.7..1.75	0.9..1.1	low
7	4.3..4.6	1.5..1.8	1.75..1.9	0.9..1.1	very-low
8	4.3..4.6	1.4..1.5	1.75..1.9	1.1..1.7	very-low

**Table 6:** Datasets.

Dataset	Cases	Number of Attributes	Concepts
Abalone	4177	8	29
Australian	690	14	2
Bankruptcy	66	5	2
Bupa	345	6	2
Connectionist Bench	208	60	2
Echocardiogram	74	7	2
Ecoli	336	8	8
Glass	214	9	6
Image Segmentation	210	19	7
Ionoshere	351	34	2
Iris	150	4	3
Leukemia	415	175	2
Pima	768	8	2
Spectrometry	25,931	15	2
Wave	512	21	3
Wine Recognition	178	13	3
Yeast	1484	8	9

**Table 7:** Error rates for three approaches to merging.

Dataset	No Merging	Scan Number	MIN Entropy	Scan Number	MAX Entropy	Scan Number
Abalone	75.58	5	-	-	-	-
Australian	13.48	1	12.61	3	13.04	1
Bankruptcy	3.03	1	-	-	-	-
Bupa	29.28	3	30.43	2	30.43	2
Connectionist Bench	16.83	1	24.04	1	24.04	1
Echocardiogram	14.86	1	14.86	2	14.86	1
Ecoli	22.02	0	17.86	0	20.54	2
Glass	24.77	3	23.36	2	23.36	2
Image Segmentation	11.90	2	-	-	13.81	0
Ionoshere	5.98	2	5.98	1	5.98	4
Iris	4.67	2	-	-	-	-
Leukemia	21.20	2	26.27	2	21.20	2
Pima	24.09	2	24.48	0	24.61	0
Spectrometry	1.13	2	1.15	5	1.19	3
Wave	23.04	1	24.02	1	23.44	3
Wine Recognition	3.93	1	3.37	1	3.37	1
Yeast	51.75	3	49.12	5	49.93	2

**Table 8:** *Z* scores for the test of differences between averages of error rates associated with three approaches to merging.

Dataset	No Merging − Merging with MIN Entropy	Scan Number	No Merging − Merging with MAX Entropy	Scan Number
Abalone	-	-	-	-
Australian	6.90	3	5.20	3
Bankruptcy	-	-	-	-
Bupa	22.90	0	12.75	0
Connectionist Bench	−9.09	1	−8.73	1
Echocardiogram	−6.71	0	−7.84	0
Ecoli	31.75	0	−140.05	0
Glass	−7.28	1	11.92	0
Image Segmentation	−0.33	0	−14.71	2
Ionoshere	−41.36	2	−8.85	3
Iris	-	-	-	-
Leukemia	−51.18	0	−45.40	0
Pima	16.34	0	27.16	2
Spectrometry	20.94	5	−14.55	3
Wave	6.92	2	10.20	3
Wine Recognition	−0.73	0	8.94	1
Yeast	25.68	2	23.14	2
